# Clinical significance and effect of AEG-1 on the proliferation, invasion, and migration of NSCLC: a study based on immunohistochemistry, TCGA, bioinformatics, *in vitro* and *in vivo* verification

**DOI:** 10.18632/oncotarget.14972

**Published:** 2017-02-01

**Authors:** Yu Zhang, Zu-Yun Li, Xin-Xi Hou, Xiao Wang, Yi-Huan Luo, Yan-Ping Ying, Gang Chen

**Affiliations:** ^1^ Department of Pathology, First Affiliated Hospital of Guangxi Medical University, Nanning, Guangxi Zhuang Autonomous Region 530021, China; ^2^ Department of Orthopedics, China-Japan Union Hospital of Jilin University, Changchun 130033, China; ^3^ Department of Nursing, The First Affiliated Hospital of Guangxi Medical University, Guangxi Zhuang Autonomous Region 530021, China

**Keywords:** astrocyte elevated gene-1 (AEG-1), non-small cell lung cancer (NSCLC), meta-analysis, TCGA, immunohistochemistry

## Abstract

**Background:**

Astrocyte elevated gene-1 (AEG-1) is related to the tumorigenesis and deterioration of different cancers, including non-small cell lung cancer (NSCLC). However, the effect of AEG-1 in NSCLC remains unclear. In this study, we aimed to investigate the clinical significance and effect of AEG-1 on biological function of NSCLC.

**Results:**

AEG-1 was significantly overexpressed in NSCLC tissues and closely correlated to the deterioration of NSCLC based on tissue microarray, TCGA database and meta-analysis. After knock-down of AEG-1, the proliferation, migration and invasion of NSCLC cells were all inhibited, and the tumorigenic and angiogenic ability of NSCLC cells were weakened. Furthermore, the AEG-1 co-expressed genes were significantly related to AMPK signaling pathway based on bioinformatics approaches.

**Materials and Methods:**

A tissue microarray, the Cancer Genome Atlas (TCGA) database, as well as a meta-analysis were performed to analyze the relationship between AEG-1 and the clinicopathological parameters of NSCLC. Furthermore, immunocytochemistry, Western blot analysis, scratch assay, colony formation assay, Transwell migration and invasion assay and the chick embryo chorioallantoic membrane (CAM) model were conducted to explore the effect of AEG-1 on NSCLC *in vitro* and *in vivo*. Additionally, bioinformatics analyses were carried out to assess the potential pathways and networks of the co-expressed genes of AEG-1.

**Conclusions:**

AEG-1 is positively activated in the tumorigenesis and deterioration of NSCLC. We hypothesize that AEG-1 could play an important role in NSCLC via AMPK signaling pathway. Inhibiting the expression of AEG-1 is expected to become a novel method in the therapeutic strategies of NSCLC.

## INTRODUCTION

Lung cancer has become the most common cancer, and the incidence rate of lung cancer is gradually growing [1–4]. About 80%–85% of the newly diagnosed lung cancers are non-small cell lung cancer (NSCLC) [5, 6]. In addition, more than 70% of the newly diagnosed NSCLC patients are advanced disease, and the 5-year survival rate of NSCLC cases is only 16% [7]. Hence, it is of great significance to further research the possible effect of NSCLC tumorigenesis and deterioration.

Astrocyte elevated gene-1 (AEG-1), also identified as Lyric (lysine-rich CEACAM1), metadherin or MTDH, was initially discovered as an HIV-1- and TNF-alpha-inducible gene in human fetal astrocytes [8]. Growing evidence reveals that AEG-1 is significantly related to the invasion, migration, apoptosis and prognosis of different cancers, such as hepatocellular carcinoma [9], bladder cancer [10], prostate cancer [11], gastric cancer [12] and so on. Zhou et al. [9] found that AEG-1 was highly expressed in high metastatic potential cancer cell lines and the AEG-1 expression was positively correlated with orientation chemotaxis and adhesion of human hepatocellular carcinoma cell lines. Wei et al. [11] found that AEG-1 could act as an oncogene and the inhibition of AEG-1 expression could promote cell apoptosis, reduce cell viability and weaken the invasive ability of prostate cancer cells. In addition, the suppression of AEG-1 expression could improve the cell sensitivity to cisplatin. Also, several studies have revealed the pivotal clinical role of AEG-1 in carcinogenesis and aggressiveness of NSCLC. For example, Lu et al. [13] found that a high AEG-1 expression was associated with the clinical stage and lymph node metastasis, and they also showed that AEG-1 could be an independent prognostic biomarker of NSCLC. Sun et al. [14] reported that the high AEG-1 expression was positively related to carcinogenesis and aggressiveness of NSCLC. However, Yao et al. [15] showed that a low AEG-1 expression was correlated with the clinical stage and lymph node metastasis of NSCLC and could provide potential in predicting metastasis and prognosis of NSCLC patients.

We designed this study to further explore the effect of AEG-1 on the proliferation, invasion and migration of NSCLC *in vitro* and *in vivo*. Firstly, we used the Cancer Genome Atlas (TCGA) database, tissue microarray, meta-analysis, and quantitative reverse transcription–polymerase chain reactions (qRT-PCRs) to assess the clinical role of AEG-1 in NSCLC. Then, scratch assay, colony formation assay, Transwell migration and invasion assays and the chick embryo chorioallantoic membrane (CAM) model were performed to clarify the possible role of AEG-1 *in vitro* and *in vivo* in NSCLC. In addition, Gene Ontology (GO), Kyoto Encyclopedia of Genes and Genomes (KEGG), and network analyses were performed to explore the potential pathways and networks of the co-expressed genes of AEG-1.

## RESULTS

### The expression of AEG-1 in a tissue microarray of NSCLC

**Figure 1:**
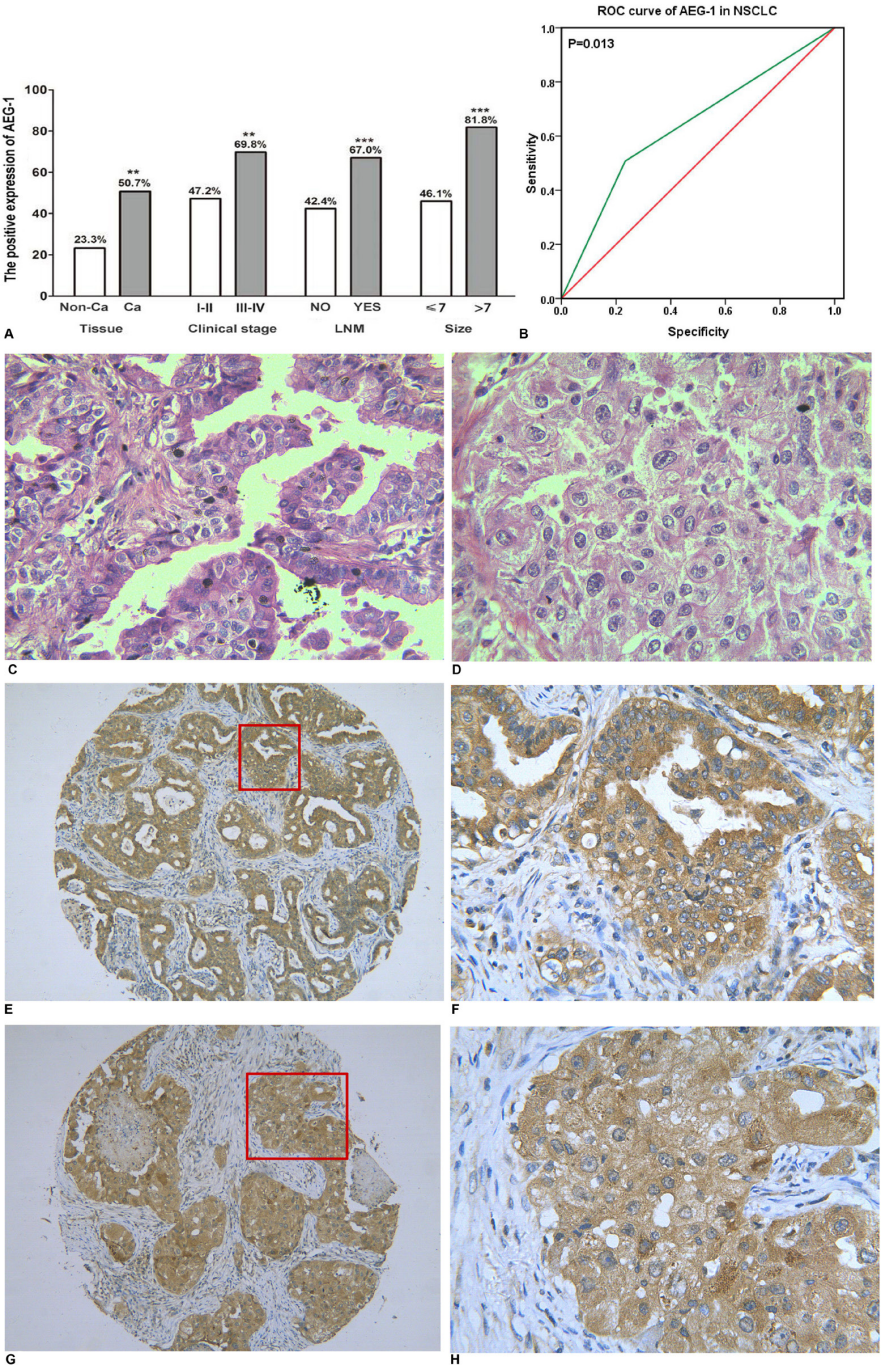
The relationship between AEG-1 expression and NSCLC (**A**) The differential expression of AEG-1 between lung cancer and normal lung tissues, and the correlation between AEG-1 expression and clinical stage, LNM and tumor size (**P < 0.01, ***P < 0.001). Note: LNM: lymph node metastasis. (**B**) ROC curve analyses of AEG-1 for predicting the clinical diagnostic value in NSCLC. The area under curve (AUC) of AEG-1 was 0.637 (95% CI 0.540–0.734, *P* = 0.013), which indicates a potential diagnostic value of AEG-1 level in NSCLC. (**C**) Hematoxylin/eosin (HE) staining of lung adenocarcinoma tissues with AEG-1 expression (x 400). (**D**) Hematoxylin/eosin (HE) staining of squamous cell carcinoma with AEG-1 expression (× 400). (**E**) Immunohistochemical staining for AEG-1 in lung adenocarcinoma (× 100). (**F**) Immunohistochemical staining for AEG-1 in lung adenocarcinoma (× 400). (**G**) Immunohistochemical staining for AEG-1 in squamous cell carcinoma (× 100). (**H**) Immunohistochemical staining for AEG-1 in squamous cell carcinoma (× 400).

As we reported previously [16], we found that AEG-1 was highly expressed in NSCLC (50.7%, *P* = 0.004) and was positively related to clinical stage (r = 0.164, *P* = 0.002), lymph node metastasis (r = 0.232, *P* < 0.001) and tumor size (r = 0.240, *P* < 0.001). The detailed results are shown in Figure 1A. Furthermore, compared to the normal lung, the expression of AEG-1 was predominantly higher in lung cancer (*P* = 0.002), including small cell lung cancer (SCLC, *P* = 0.004) and NSCLC (*P* = 0.004, Table 1). We also investigated the AEG-1 expression in the distinct histologic types of NSCLC. We found that AEG-1 had a remarkably higher expression in adenocarcinoma (*P* = 0.002), squamous cell carcinoma (*P* = 0.008), adenosquamous carcinoma (*P* = 0.008), and undifferentiated carcinoma (*P* = 0.035) compared to the normal lung (Table 1). Next, we further investigated the clinical contribution of AEG-1 in the subtypes (lung adenocarcinoma and squamous cell carcinoma) of NSCLC. In lung adenocarcinoma, the expression of AEG-1 was closely related to clinical stage (*P* = 0.016), tumor size (*P* < 0.001) and lymph node metastasis (*P* = 0.009, Table 2). With regard to squamous cell carcinoma, the consistent associations between AEG-1 expression and tumor size (*P* = 0.007) and lymph node metastasis (*P* = 0.010, Table 3.) were also found. Additionally, the relationship between AEG-1 expression and the clinical diagnostic value was analyzed by a receiver operating characteristic (ROC) curve. The area under curve (AUC) of AEG-1 was 0.637 (95% CI 0.540–0.734, *P* = 0.013), which indicate a potential diagnostic value of AEG-1 level in NSCLC (Figure 1B).

**Table 1:** Expression of AEG-1 protein in lung cancer and normal lung

Tissue	Case	AEG-1 positive	AEG-1negative	Z	*P*
**Normal lung tissue**	30	7	23		
**Lung cancer**	365	188	177	3.403	0.002
**Histology**					
**SCLC**	26	16	10	3.055	0.004
**NSCLC**	339	172	167	2.904	0.004
**Adenocarcinoma**	127	68	59	3.348	0.002
**Squamous cell carcinoma**	175	83	92	2.764	0.008
**Adenosquamous carcinoma**	28	16	12	2.739	0.008
**Undifferentiated carcinoma**	8	5	3	2.195	0.035
**Large cell carcinoma**	1	0	1	–	–

Note: SCLC: small cell lung cancer.

NSCLC: non- small cell lung cancer.

**Table 2:** Differential expression of the AEG-1 protein and other clinicopathological parameters in lung adenocarcinoma

Tissue	Case	AEG-1 positive	AEG-1 negative	Z	*P*
**Age(years)**				0.883	0.379
**< 60**	70	35	35		
**≥60**	57	33	24		
**Gender**				–0.775	0.440
**Male**	82	46	36		
**Female**	45	22	23		
**Clinical stage**				2.536	0.016
I **or II**	106	52	54		
**III or IV**	21	16	5		
**Tumor size (cm)**				4.438	< 0.001
**≤7**	105	49	56		
**> 7**	22	19	3		
**Distal metastasis**				–1.693	0.122
**Yes**	9	7	2		
**No**	118	61	57		
**Lymph node metastasis**				–2.669	0.009
**Yes**	45	31	14		
**No**	82	37	45		

**Table 3:** Differential expression of the AEG-1 protein and other clinicopathological parameters in squamous cell carcinoma

Tissue	Case	AEG-1 positive	AEG-1 negative	Z	*P*
**Age(years)**				0.396	0.693
**< 60**	85	39	46		
**≥ 60**	90	44	46		
**Gender**				1.338	0.183
**Male**	148	67	81		
**Female**	27	16	11		
**Clinical stage**				1.338	0.183
I **or II**	148	67	81		
**III or IV**	27	16	11		
**Tumor size (cm)**				2.907	0.007
**≤7**	155	68	87		
**> 7**	20	15	5		
**Distal metastasis**				–0.523	0.602
**Yes**	7	4	3		
**No**	168	79	89		
**Lymph node metastasis**				–2.608	0.010
**Yes**	57	35	22		
**No**	118	48	70		

Thus, immunohistochemistry was used to detect the expression of AEG-1. The positive signaling of AEG-1 was located in the periphery of the nucleus or cytoplasm of the NSCLC cells via immunohistochemical staining. The positive cells showed a diffuse brown-yellow or dark brown color (Figure 1C–1H). Among all of the 339 cases of NSCLC, 172 cases were AEG-1 positive (50.7%), which was significantly higher than that in the normal lung tissues (23.3%, *P* = 0.004).

### Supplementary information from The Cancer Genome Atlas (TCGA) database

**Figure 2:**
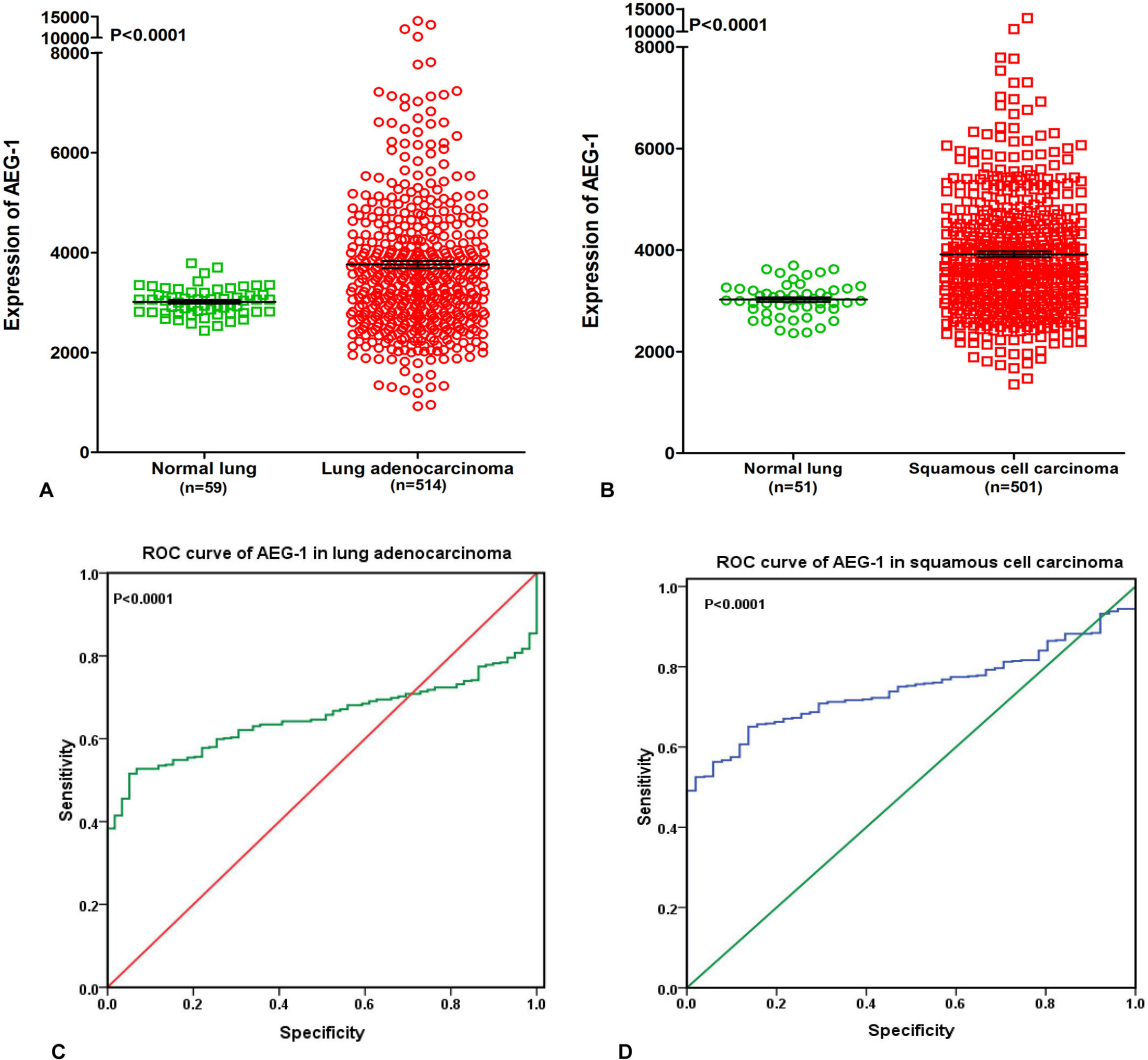
Supplementary information from The Cancer Genome Atlas (TCGA) database (**A**) AEG-1 was highly expressed in lung adenocarcinoma compared to the non-cancerous lung tissues based on The Cancer Genome Atlas (TCGA) database (*P* < 0.0001). (**B**) AEG-1 was highly expressed in squamous cell carcinoma compared to the non-cancerous lung tissues based on The Cancer Genome Atlas (TCGA) database (*P* < 0.0001). (**C**) ROC curve analyses of AEG-1 for predicting the clinical diagnostic value in lung adenocarcinoma patients based on The Cancer Genome Atlas (TCGA) database. The area under curve (AUC) of AEG-1 was 0.650 (95%CI 0.607∼0.694, *P* < 0.0001), which indicates a certain diagnostic value of AEG-1 level in lung adenocarcinoma. (**D**) ROC curve analyses of AEG-1 for predicting the clinical diagnostic value in squamous cell carcinoma patients based on The Cancer Genome Atlas (TCGA) database. The area under curve (AUC) of AEG-1 was 0.746 (95%CI 0.702∼0.789, *P* < 0.0001), which indicates a potential diagnostic value of AEG-1 level in squamous cell carcinoma.

To further elucidate the relationship between AEG-1 and NSCLC, we performed a clinical study with the original data in TCGA. We found that AEG-1 was highly expressed in both lung adenocarcinoma and squamous cell carcinoma compared to the non-cancerous lung tissues (*P* < 0.0001, Figure 2A, 2B). We also investigated the relationship between AEG-1 and the clinical parameters of NSCLC, and we found that the high expression of AEG-1 was weakly related to gender (*P* = 0.042). We then searched the PubMed to further explore the relationship between AEG-1 and gender or hormone level, but no positive associations were found. Additionally, we found that the AUC of AEG-1 was 0.650 (95%CI 0.607∼0.694, *P <* 0.0001) for the lung adenocarcinoma patients and was 0.746 (95%CI 0.702∼0.789, *P* < 0.0001) for the squamous cell carcinoma patients, which could gain moderate diagnostic value of AEG-1 level in NSCLC (all *P* < 0.0001, Figure 2C, 2D). Furthermore, we also studied the relationships between AEG-1 expression and other clinicopathological parameters in lung cancer, but no obvious associations were found based on the TCGA database.

### AEG-1 and NSCLC: a meta-analysis

**Figure 3:**
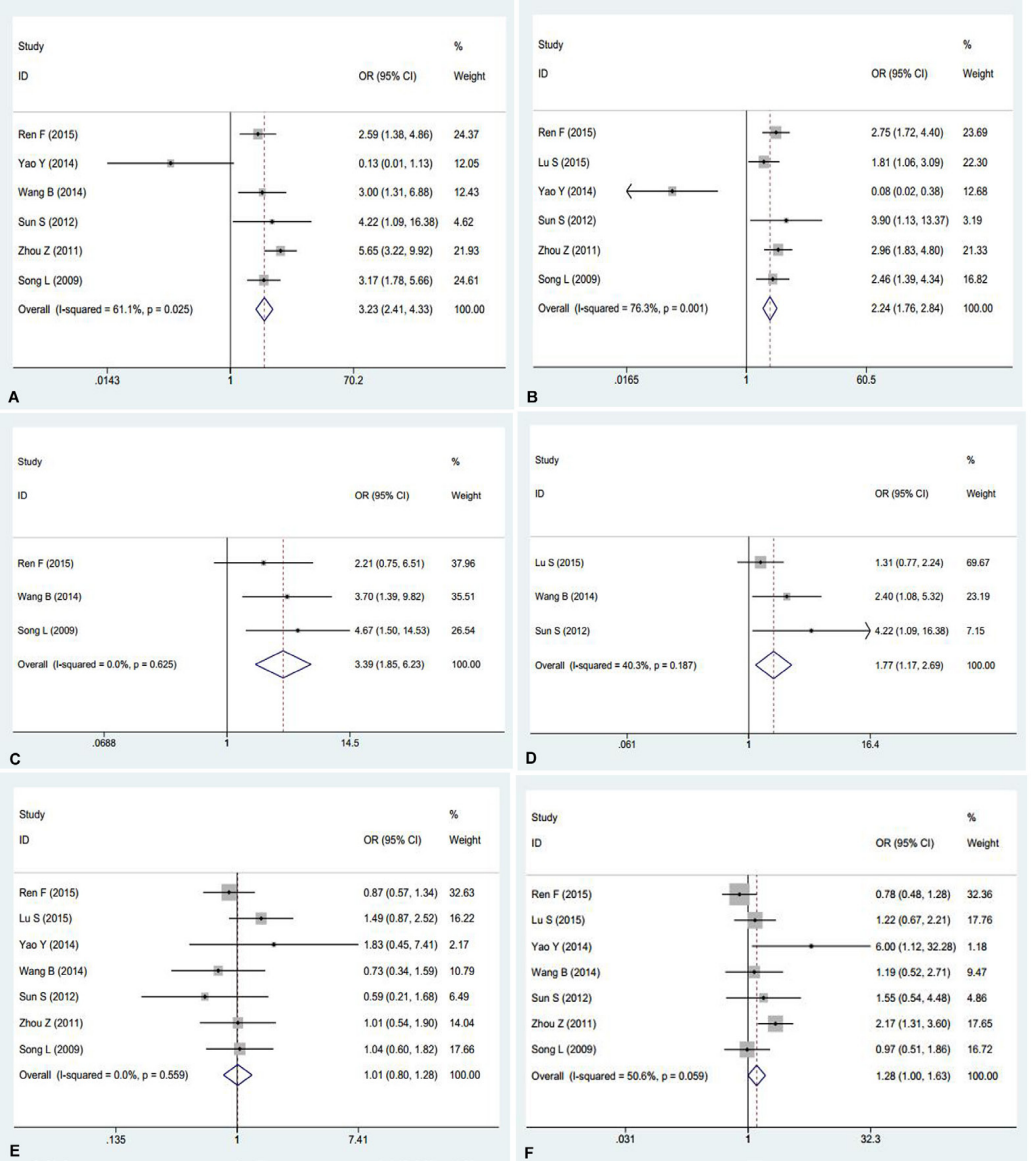
Meta-analysis evaluating the relationships between AEG-1 expression and clinicopathological parameters in patients with NSCLC (**A**) Clinical stage (OR=3.23, 95%CI: 2.41–4.33, *P* < 0.001). (**B**) Lymph node metastasis (OR = 2.24, 95%CI: 1.76–2.84, *P* < 0.001). (**C**) Distant metastasis (OR=3.39, 95%CI: 1.85–6.23, *P* < 0.001). (**D**) Histological differentiation (OR = 1.77, 95%CI: 1.17–2.69, *P* = 0.007). (**E**) Age (OR = 1.01, 95%CI: 0.80–1.28, *P* = 0.937).(**F**) Gender (OR =1.28, 95%CI: 1.00–1.63, *P* = 0.054).

AEG-1 has been reported to relate to the tumorigenesis and deterioration of different cancers. However, a thorough summary of all of the published studies on AEG-1 in NSCLC still lacks. Consequently, we performed this meta-analysis to provide readers with direct information on the clinical effects of AEG-1 in NSCLC. In the end, 7 eligible studies [13–19], including our previous studies [16], were selected. The characteristics of studies included in this meta-analysis were outlined in Table 4. Furthermore, the current meta-analysis included 1303 patients, and our results suggested that strong relations emerged between the high expression of AEG-1 and clinical stage (OR=3.23, 95%CI: 2.41–4.33, *P* < 0.001), lymph node metastasis (OR=2.24, 95%CI: 1.76–2.84, *P* < 0.001), distant metastasis (OR=3.39, 95%CI: 1.85–6.23, *P* < 0.001), and histological differentiation (OR=1.77, 95%CI: 1.17–2.69, *P* = 0.007), but does not support associations between age (OR=1.01, 95%CI: 0.80–1.28, *P* = 0.937) or gender (OR=1.28, 95%CI: 1.00–1.63, *P* = 0.054) and a high expression of AEG-1 (Figure 3). Additionally, a high expression of AEG-1 was pronouncedly related to a poor overall survival (OS) (HR=1.82, 95%CI: 1.35–2.28, *P* < 0.0001).

**Table 4:** Characteristics of studies included in this meta-analysis

First author	Year	Origin	Sample size (case)	Quantitative method	Language
Ren F	2015	China	339	IHC	English
Lu S	2015	China	225	IHC	English
Yao Y	2014	China	40	IHC	English
Wang B	2014	China	104	RT-PCR	Chinese
Sun S	2012	China	67	IHC	English
Zhou Z	2011	China	328	IHC	Chinese
Song L	2009	China	200	IHC	English

Note: IHC: immunohistochemistry;

RT-PCR: reverse transcription-Polymerase Chain Reaction.

**Figure 4:**
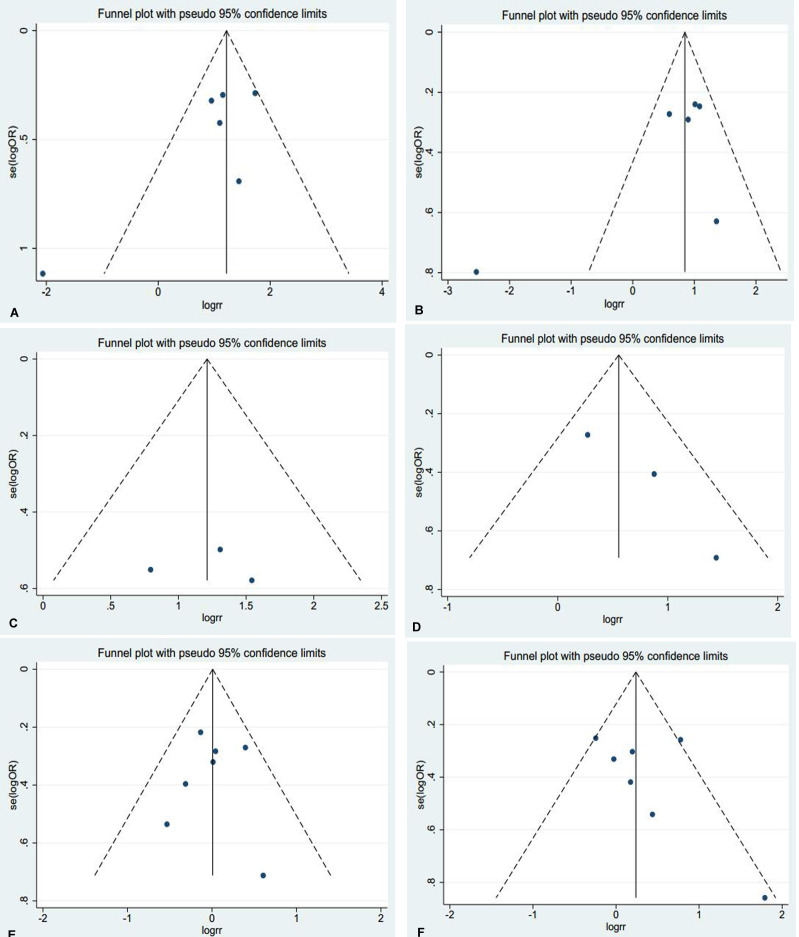
Funnel plots were used to estimate potential publication bias between the different clinical parameters and AEG-1 (Begg's method was employed) (**A**) Clinical stage (**B**) Lymph node metastasis (**C**) Distant metastasis (**D**) Histological differentiation (**E**) Age (**F**) Gender.

**Figure 5:**
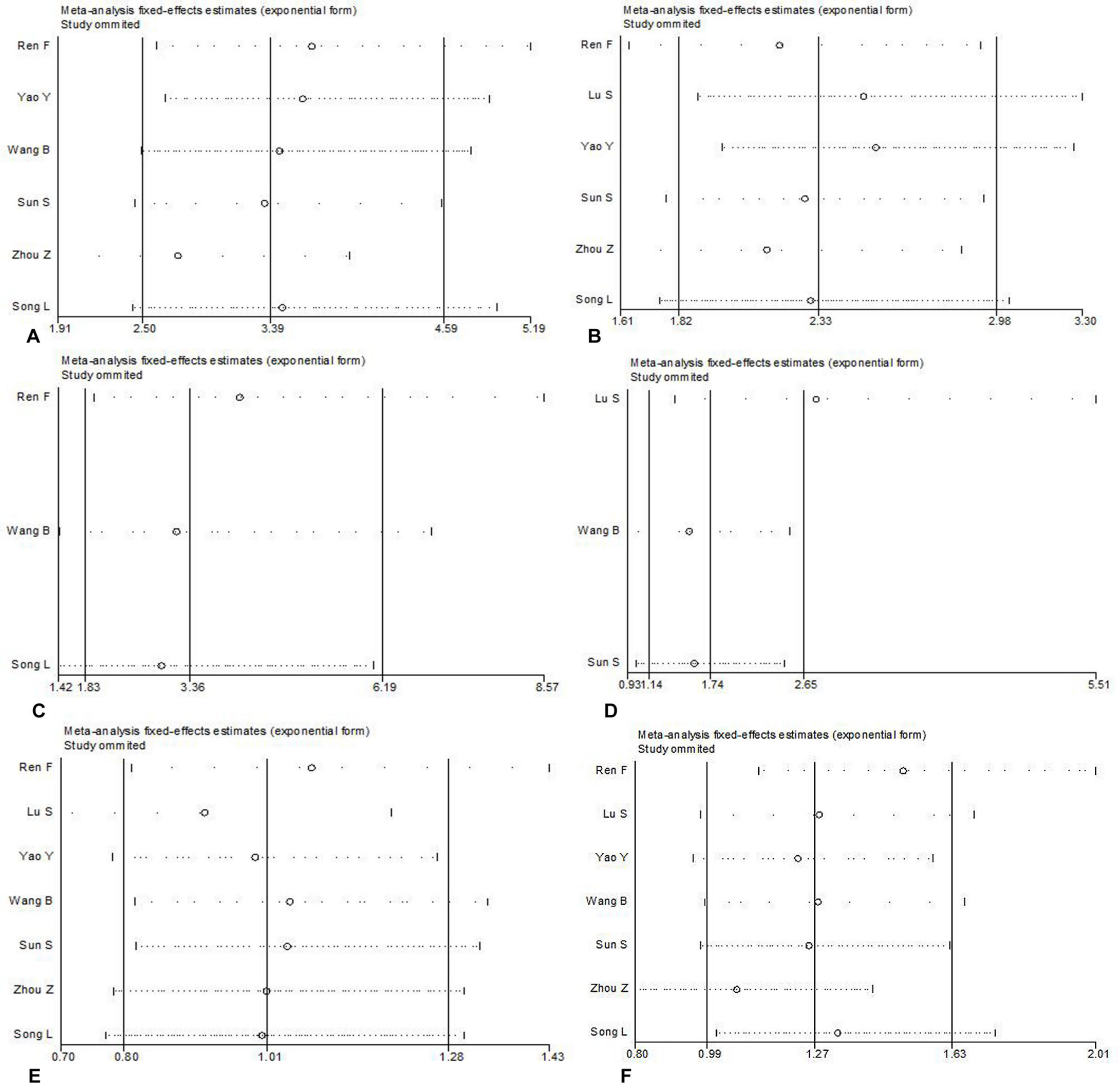
Sensitivity analysis was performed to exclude one study at a time to estimate whether the results were just due to a study with an extreme result or a large number (**A**) Clinical stage (**B**) Lymph node metastasis (**C**) Distant metastasis (**D**) Histological differentiation (**E**) Age (**F**) Gender.

Thus, Begg's or Egger's test was used to access the publication bias of the included articles. In this meta-analysis, the funnel plot was symmetrical, and no publication bias was found (all *P* > 0.05, Figure 4). However, we found that, among the significant subgroups, heterogeneity existed in the clinical stage (I^2^ (%)=61.1%, *P* = 0.025) and lymph node metastasis (I^2^ (%)=76.3%, *P* = 0.001). This might be a limitation of our meta-analysis partly because of the null findings of some studies, especially those studies with small sample sizes or opposite conclusions. To explore whether the results were just due to a study with an extreme result or a large number, a sensitivity analysis was performed to exclude one study at a time. Based on the results of the sensitivity analysis, we found that no studies had an obvious influence on the summary estimate (Figure 5). To further investigate the sources of heterogeneity, Yao^’^s study was removed because its conclusions were the opposite of others. After that, we found that obvious associations emerged between the high expression of AEG-1 and the clinical stage (OR = 3.66, 95%CI: 2.70–4.96, I^2^ = 0%) and lymph node metastasis (OR = 2.55, 95%CI: 1.99–3.27, I^2^=0%), indicating that Yao^’^s study might contribute to the high heterogeneity in the clinical stage and lymph node metastasis subgroups.

### The expression of AEG-1 in different cell lines of NSCLC

**Figure 6:**
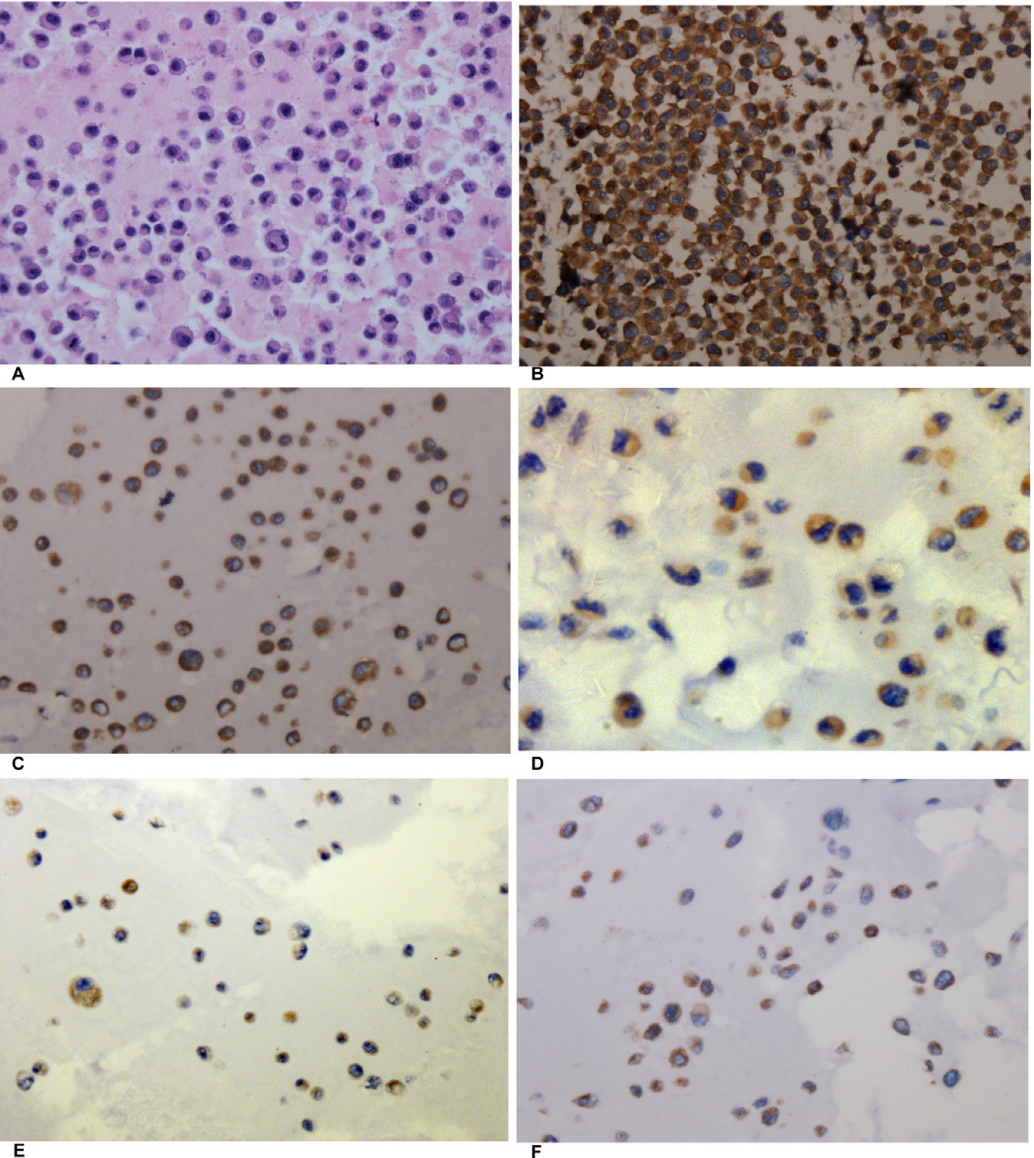
The expression of AEG-1 in different cell blocks of NSCLC cell lines (**A**) Hematoxylin/eosin (HE) staining of the LM3 cell line of hepatocellular carcinoma with AEG-1 expression (× 400, control). (**B**) Immunohistochemical staining of the LM3 cell line of hepatocellular carcinoma with AEG-1 expression (× 400, control). (**C**) Immunohistochemical staining for AEG-1 in the H460 cell line of NSCLC (× 400). (**D**) Immunohistochemical staining for AEG-1 in the H1299 cell line of NSCLC (× 400). (**E**) Immunohistochemical staining for AEG-1 in the A549 cell line of NSCLC (× 400). (**F**) Immunohistochemical staining for AEG-1 in the PC9 cell line of NSCLC (× 400).

In this study, four human NSCLC cell lines (H460, H1299, A549, and PC9) were cultured, and cell block immunohistochemistry was used to assess the expression intensity of AEG-1. The results of cell block immunohistochemistry showed that AEG-1 was differentially expressed in the H460, H1299, A549 and PC9 cell lines (Figure 6). Among these four NSCLC cell lines, H460 showed the highest expression intensity with a positive rate of 80%. The positive rate of the H1299, A549 and PC9 cell lines was 60%, 40%, and 40%, respectively.

**Figure 7:**
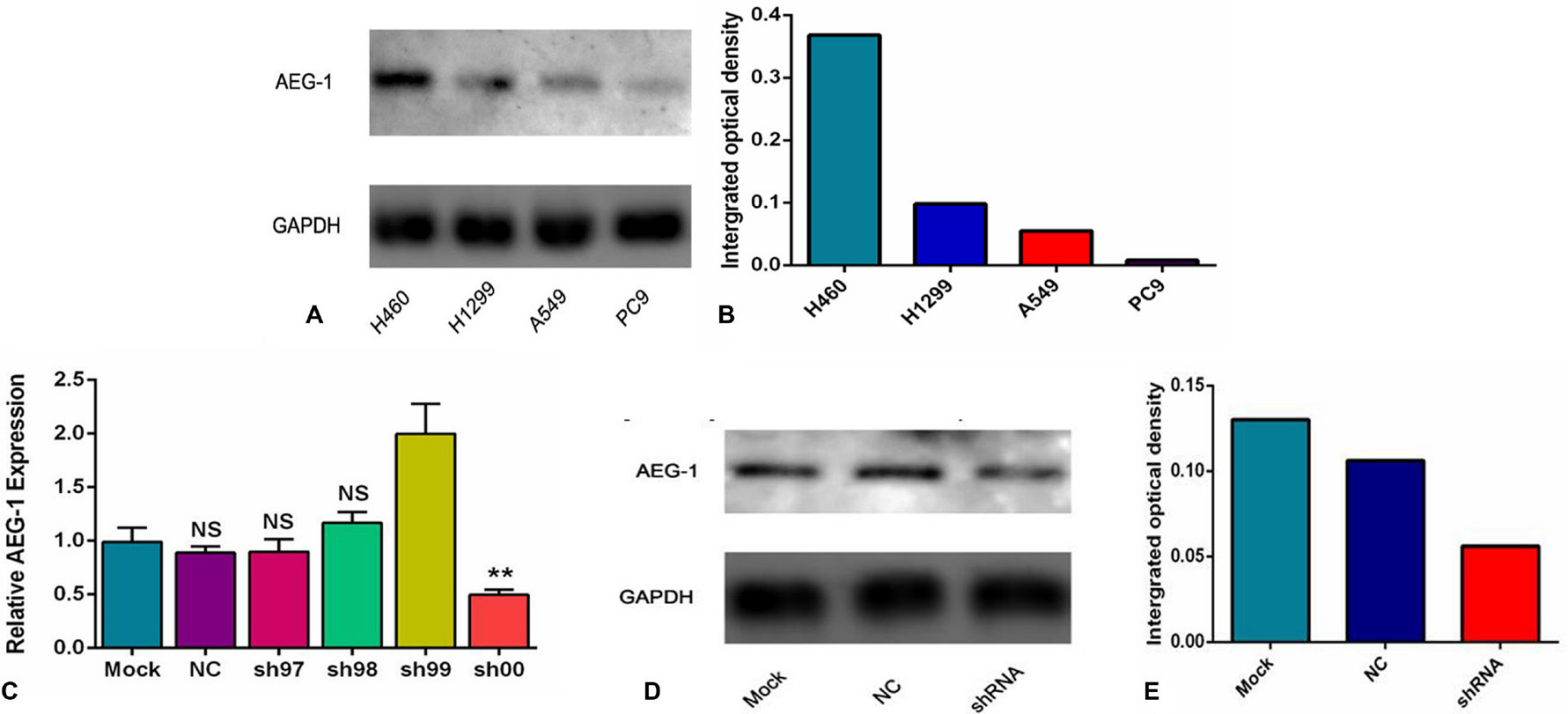
The expression intensity of AEG-1 protein in different cell lines and groups (**A**) The expression of AEG-1 protein in different cell lines of NSCLC was detected by a Western blot at the protein level. (**B**) The integrated optical density (IOD) value of the AEG-1 protein in the different cell lines of NSCLC. (**C**) The expression of AEG-1 was silenced in the different groups at the transcription level. (**P < 0.01). (**D**) The expression of AEG-1 protein in the different groups was detected by a Western blot at the protein level. (**E**) The integrated optical density (IOD) values of AEG-1 protein in the different groups.

Furthermore, to further evaluate the expression intensity of AEG-1 protein, a Western blot analysis was performed. AEG-1 protein expression was highest in the H460 cells with the integrated optical density (IOD) value of 0.368. The IOD value of the H1299, A549 and PC9 cell lines was 0.098, 0.055, and 0.008, respectively (Figure 7A, 7B). According to the aforementioned results, the H460 cell line was selected to do further research in this study.

### The transfection with shRNA plasmid

In this study, to further explore the role of AEG-1 in NSCLC, we aimed to silence the expression of AEG-1 in 6 groups (mock control, NC, sh97, sh98, sh99 and sh00) at the transcription and protein level. We found that the expression of AEG-1 in the sh00 group (0.50 ± 0.04) was obviously inhibited compared to the other groups (*P* = 0.005, mock control, NC, sh97, sh98 and sh99 was 0.99 ± 0.13, 0.88 ± 0.06, 0.90 ± 0.11, 1.17 ± 0.10 and 1.99 ± 0.28, respectively. Figure 7C) at the transcription level. Consistent with the results of the transcription level, Western blot showed that AEG-1 was silenced in the shRNA group with an IOD value of 0.056 (Figure 7D, 7E) at protein level, and the IOD values of the mock control and the NC were 0.13 and 0.106, respectively. Based on these results, the sh00 group was selected for further research in this study.

**Figure 8:**
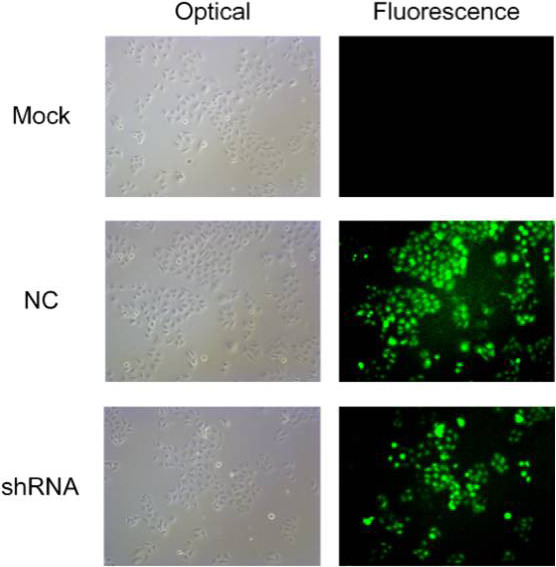
The immunofluorescence images of the AEG-1 staining in the different groups ShRNA plasmids were transfected into three groups (mock control, NC and shRNA). The results of immunofluorescence images show that the expression of AEG-1 was inhibited in the shRNA group with the transfection rate being approximately 80%.

Next, we further investigated the inhibition with AEG-1-shRNA. Sh00 plasmids were transfected into three groups (mock control, NC and shRNA). The immunofluorescence images of the AEG-1 staining in the different groups are shown in Figure 8. The results showed that the expression of AEG-1 was inhibited in the shRNA group with the transfection rate being approximately 80%.

### Proliferation inhibited by AEG-1-shRNA

**Figure 9:**
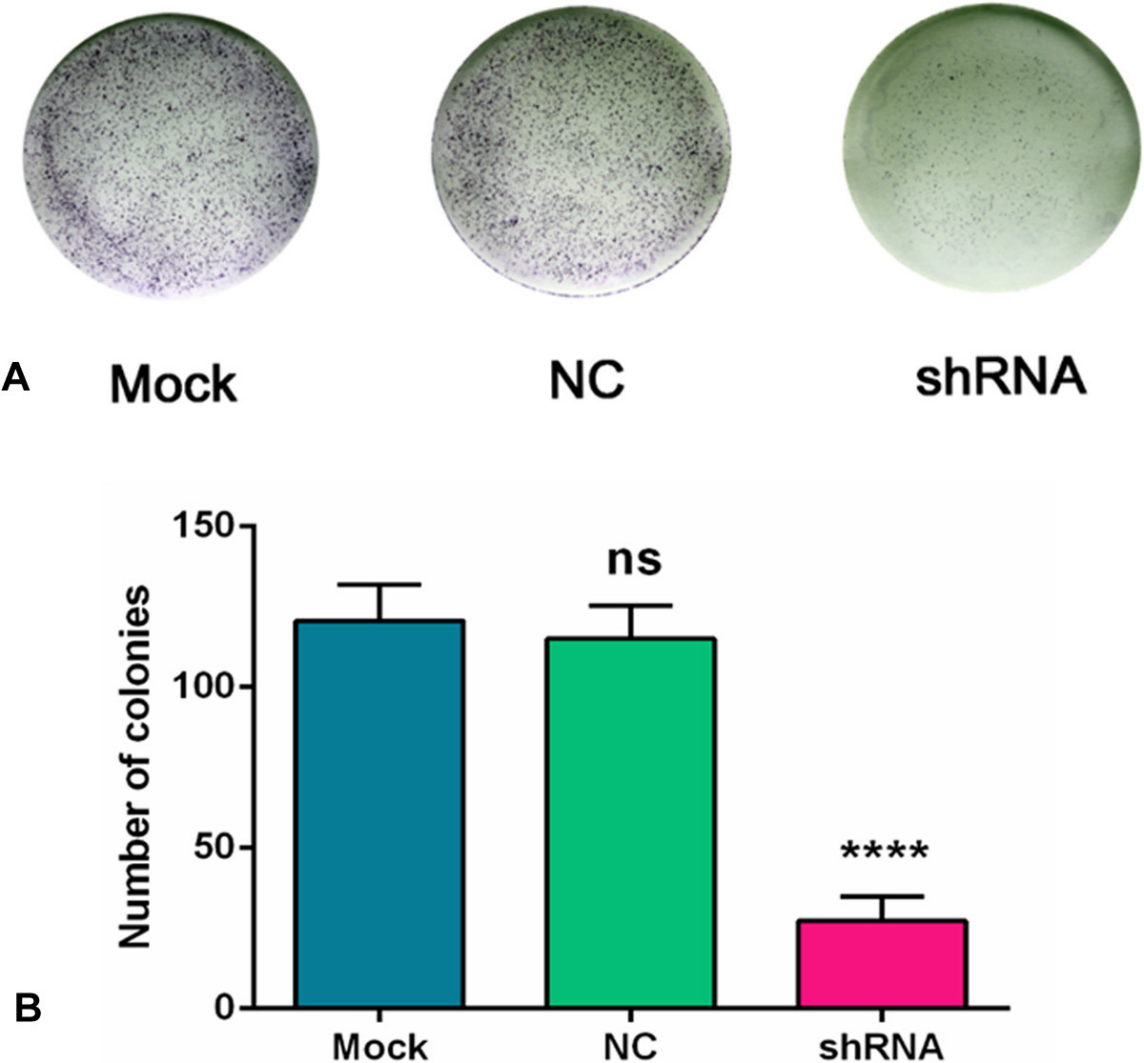
AEG-1-shRNA inhibited the proliferation of H460 cells (**A**) A colony formation assay was performed to evaluate the proliferation of the H460 cells. A colony was counted if the number of cells was more than 10. The results showed that a large cell proliferation inhibition was observed in the H460 cells, especially in the AEG-1-shRNA group. (**B**) The number of colonies in the mock, NC and AEG-1-shRNA groups were counted. The graph represents the Mean ± SD. (****P < 0.0001).

A colony formation assay was performed to evaluate the proliferation of the H460 cells. A colony was counted if the number of cells was more than 10. The results showed that a large cell proliferation inhibition was observed in the H460 cells, especially in the AEG-1-shRNA group. The number of colonies in the mock, NC and AEG-1-shRNA groups was 120.70 ± 11.24, 115.70 ± 10.39 and 27.33 ± 7.63, respectively, indicating that AEG-1 obviously improved the proliferation of the H460 cells (*P* < 0.0001, Figure 9).

### Cell migration inhibitory effect of AEG-1-shRNA

**Figure 10:**
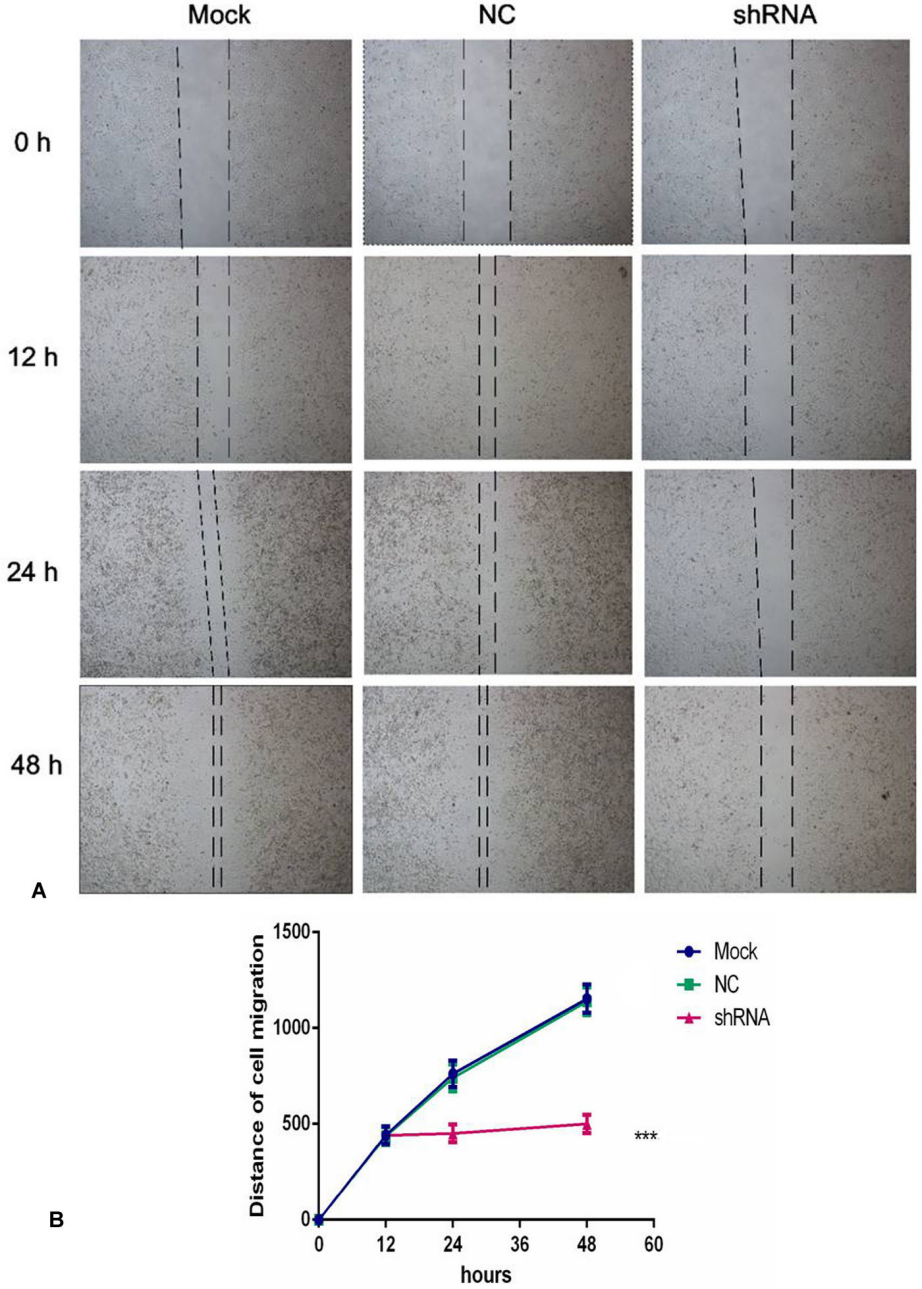
AEG-1-shRNA inhibited the migration of the H460 cells (**A**) A scratch assay was performed to further observe the effect of the AEG-1-shRNA on the migration of H460 cells. And the migration ability was significantly inhibited after treatment with the AEG-1-shRNA compared with the mock and NC groups. (**B**) The wound width of the mock, NC and AEG-1-shRNA groups. The graph represents the Mean ±SD. (**P < 0.01, ***P < 0.0001).

To further observe the effect of the AEG-1-shRNA on the migration of NSCLC, we performed a scratch assay using the H460 cells. The results showed that the migration ability was significantly inhibited after treatment with the AEG-1-shRNA compared with the mock and NC groups (Figure 10). Thus, after transfecting with the AEG-1-shRNA for 48 h, a significant difference was found among the AEG-1-shRNA (550 ±139.30), NC (1139.33 ±217.26), and mock groups (1153 ±219.45), indicating that AEG-1 significantly promotes the migration of the H460 cells. The migration distance of the NSCLC cell lines in MOCK, NC and shRNA groups at 0 h, 12 h, 24 h and 48 h was shown in Table 5. Furthermore, a Transwell migration assay was used to further verify the results of the scratch assay. Consistent with the scratch assay, the migration ability in the AEG-1-shRNA group was remarkably inhibited in the H460 cells compared to the control group (data not shown). Thus, we can conclude that AEG-1 significantly promotes the migration of NSCLC cells.

**Table 5:** The migration distance of the NSCLC cell lines in the different groups in the scratch assay

Time(h)	0 h	12 h	24 h	48 h
Group				
Mock	0	441 ±137.88	761.66 ±205.94	1153 ±219.45
NC	0	431.44 ±138.43	740.11 ±212.34	1139.33 ±217.26
shRNA	0	440.55 ±138.72	450.88 ±137.97	550 ±139.30

NC group: negative shRNA control group.

### Contribution of AEG-1-shRNA to cell invasion

**Figure 11:**
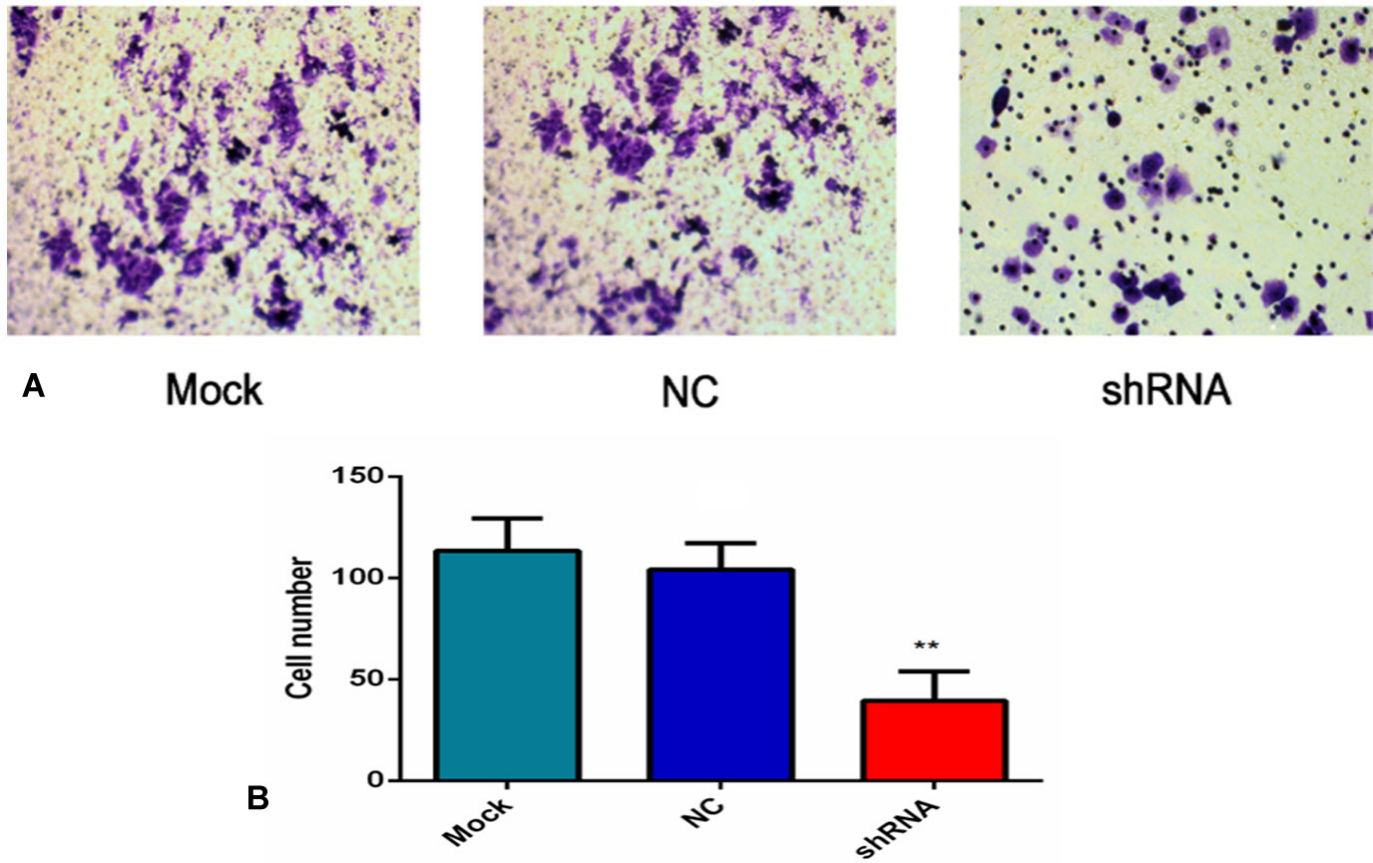
AEG-1-shRNA inhibited the invasion of the H460 cells (**A**) Transwell invasion assay was performed to explore whether the expression of AEG-1 affected cell invasion, and the least number of the migratory cells was found in the shRNA group. (*P* < 0.01). (**B**) The number of invasive cells in the mock, NC and AEG-1-shRNA groups were counted. The graph represents the Mean ±SD. (**P < 0.01).

As shown in Figure 11A, the number of the invasive cells was diverse in the three groups. Specifically, the number of the invasive cells in the mock and NC groups (113.33 ±16.07 and 104 ±13.08) was obviously more than AEG-1-shRNA group (39.33 ±14.57). These results demonstrate that AEG-1 contributes to the invasion activity of the H460 cells (*P* < 0.01, Figure 11B).

### Effect of AEG-1-shRNA on tumorigenesis and angiogenesis by a CAM model of NSCLC

**Figure 12:**
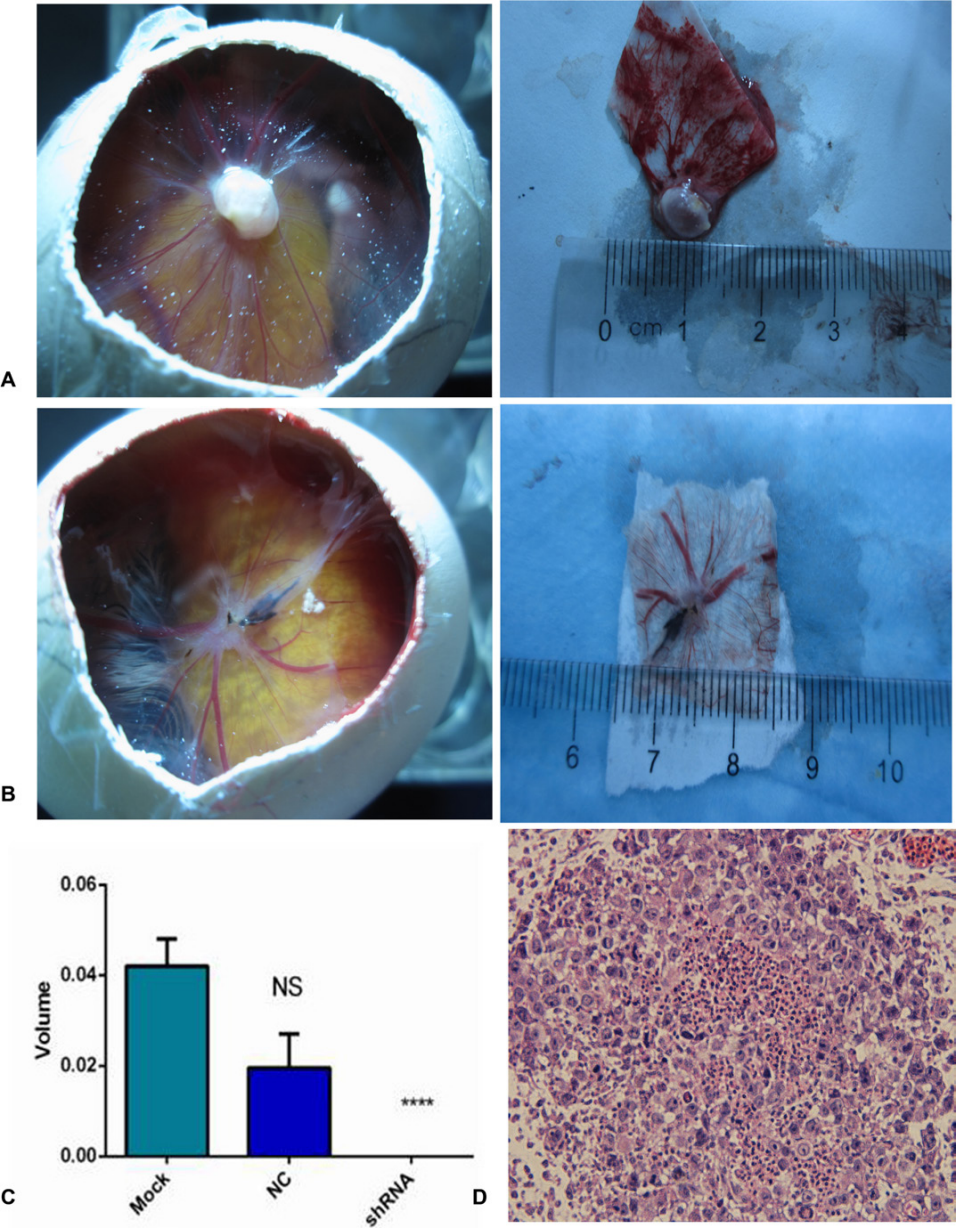
AEG-1-shRNA inhibited the tumorigenic and angiogenic ability of the H460 cells (**A**) Tumor xenografts was formed in chorioallantoic membranes (CAMs) in MOCK group. After removing the xenografts, the size was measured. (**B**) Tumor xenografts was formed in chorioallantoic membranes (CAMs) in NC group. After removing the xenografts, the size was measured. (**C**) The tumorigenic ability of the mock, NC and AEG-1-shRNA groups. The graph represents the Mean ±SD. (****P < 0.0001) (**D**) Hematoxylin/eosin (HE) staining of the tumor xenografts (× 400).

In this study, we constructed an *in vivo* CAM model to explore NSCLC tumor biology. At 5 days after inoculation with H460 cells, new blood vessels were induced and the CAMs were cautiously removed and observed under confocal microscopy (Figure 12A, 12B). The size of the tumor xenografts in the mock, NC and AEG-1-shRNA groups was 0.04 ±0.01, 0.02 ±0.01 and 0 cm^3^, respectively, indicating that the tumorigenic ability of the H460 cells was weakened by silencing AEG-1 expression (*P* < 0.05, Figure 12C).

Moreover, the hematoxylin/eosin (HE) staining of the derived xenografts showed that undifferentiated tumor cells with large nuclei were distributed in the CAM membrane surface and the chorioallantoic mesenchymal. Furthermore, tumor cell nests were also present in the membrane stroma (Figure 12D).

### The potential pathways associated with AEG-1

**Figure 13:**
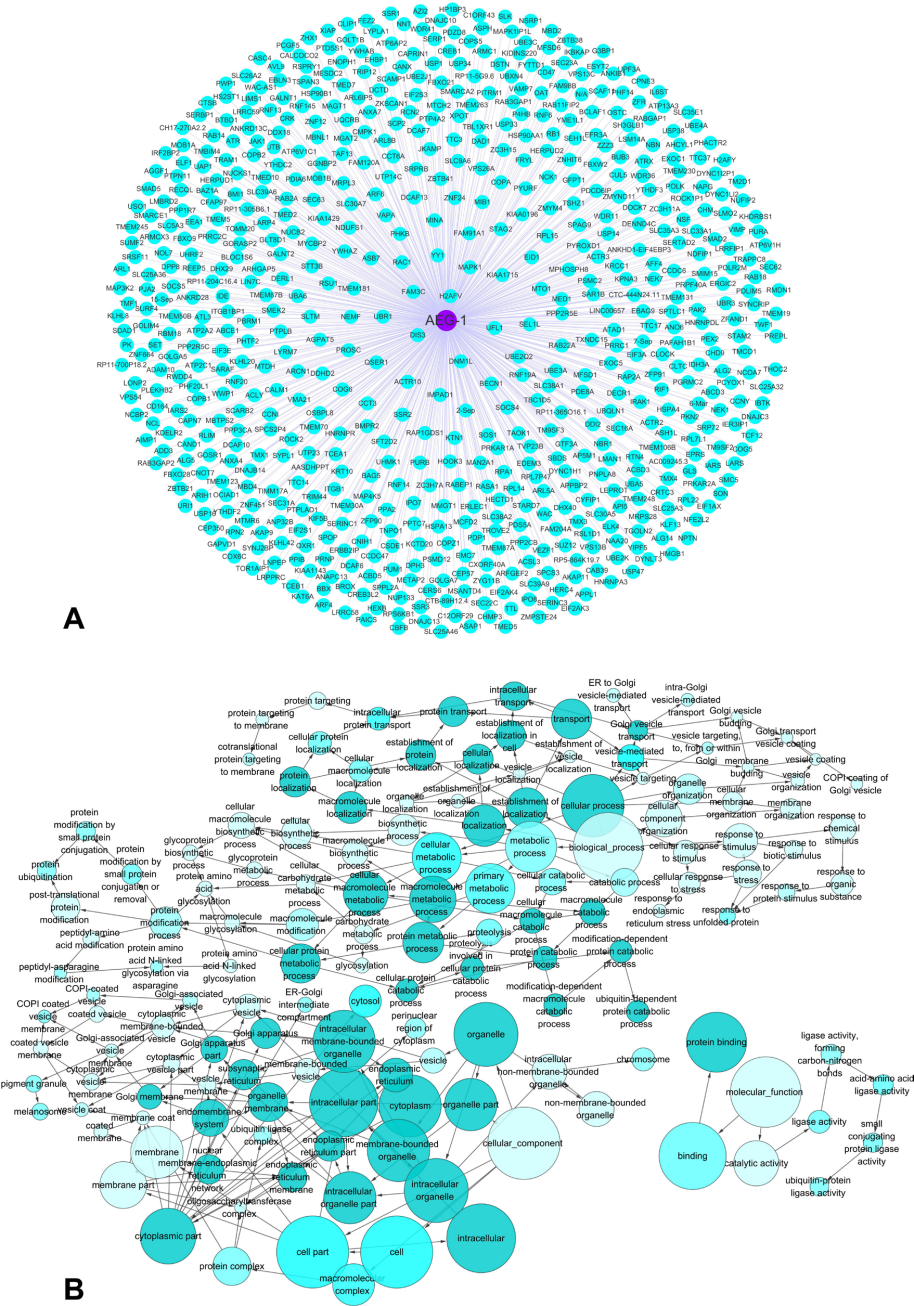
The co-expressed genes of AEG-1 and AEG-1-associated biological functions based on MEM and the DAVID Bioinformatics Tool (**A**) The network of 658 co-expressed genes of AEG-1 overlapping in five probe sets (212248_AT, 212250_AT, 212251_AT, 227277_AT and 1559822_S_At). (**B**) A function network of Gene Ontology (GO) terms for the co-expressed genes of AEG-1 in NSCLC.

A silico analysis was applied to explore AEG-1-associated biological functions and pathways based on MEM and the DAVID Bioinformatics Tool. We searched for top 2000 co-expressed genes in five different probe sets for AEG-1 (212248_AT, 212250_AT, 212251_AT, 227277_AT and 1559822_S_At). Among these co-expressed genes, 658 genes were selected that were predicted by more than four probe sets, and these genes were used for GO and pathway analyses (Figure 13A). The most strongly enriched functional terms were revealed as following: protein transport, response to endoplasmic reticulum stress, protein binding and ubiquitin-protein transferase activity. To further elucidate the functions of these co-expressed genes, a function network was constructed based on the GO analysis (Figure 13B). Besides, the KEGG pathway analysis revealed that AEG-1 co-expressed genes were significantly overrepresented in protein processing in endoplasmic reticulum, protein export, RNA transport and AMPK signaling pathway. The top five most significant GO terms (BP, CC and MF) and the top ten KEGG pathway items were present in Table 6 and Table 7. Altogether, the GO terms and KEGG pathway items provided evidences that AEG-1 might be involved in the biological mechanism of NSCLC.

**Table 6:** Top 5 enrichment GO terms (BP, CC, and MF) of the co-expressed genes of AEG-1

GO ID	Term	Ontology	Count	*P* value
GO:0006888	ER to Golgi vesicle-mediated transport	BP	31	2.68E-14
GO:0015031	protein transport	BP	45	8.66E-12
GO:0034976	response to endoplasmic reticulum stress	BP	17	4.23E-09
GO:0030433	ER-associated ubiquitin-dependent protein catabolic process	BP	14	8.06E-08
GO:0006511	ubiquitin-dependent protein catabolic process	BP	23	3.28E-07
GO:0016020	membrane	CC	171	2.34E-27
GO:0005789	endoplasmic reticulum membrane	CC	88	1.37E-20
GO:0005783	endoplasmic reticulum	CC	77	8.25E-16
GO:0005829	cytosol	CC	187	3.17E-13
GO:0000139	Golgi membrane	CC	54	5.95E-11
GO:0005515	protein binding	MF	296	2.50E-15
GO:0044822	poly(A) RNA binding	MF	86	9.23E-13
GO:0004842	ubiquitin-protein transferase activity	MF	33	6.46E-08
GO:0098641	cadherin binding involved in cell-cell adhesion	MF	29	5.00E-07
GO:0016874	ligase activity	MF	26	6.43E-06

Note: GO, Gene Ontology.

BP: biological process

CC: cellular component

MF: molecular function

**Table 7:** KEGG pathway enrichment analysis of the co-expressed genes of AEG-1

KEGG ID	KEGG term	Count	*P* value
hsa04141	Protein processing in endoplasmic reticulum	32	1.94E-15
hsa04120	Ubiquitin mediated proteolysis	14	5.32E-04
hsa00510	N-Glycan biosynthesis	8	9.97E-04
hsa03060	Protein export	5	0.006141
hsa04114	Oocyte meiosis	10	0.009383
hsa04144	Endocytosis	17	0.01063
hsa05211	Renal cell carcinoma	7	0.019344
hsa03013	RNA transport	12	0.025851
hsa04550	Signaling pathways regulating pluripotency of stem cells	10	0.040536
hsa04152	AMPK signaling pathway	9	0.04707

Notes: KEGG, Kyoto Encyclopedia of Genes and Genomes.

## DISCUSSION

In our current study, we utilized the TCGA database, a meta-analysis and experiments *in vitro* and *vivo* to further explore the clinical significance and effect of AEG-1 in NSCLC. As a result, AEG-1 was significantly overexpressed in NSCLC tissues based on TCGA database. Furthermore, we used ROC curve to assess the association between AEG-1 expression and the clinical diagnostic value, and the AUC of AEG-1 indicates a potential diagnostic value of AEG-1 level in NSCLC. Moreover, we are the first one to perform a meta-analysis on AEG-1 expression in NSCLC as further to support the clinical findings in our present study. In this meta-analysis, we compared the difference of AEG-1 between clinical stage I and II and clinical stage III and IV, between with lymph node metastasis and without lymph node metastasis, between with distant metastasis and without distant metastasis, as well as between histological differentiation (Poorly) and histological differentiation (Well/Moderately), and the results may suggest the possibility of using the expression level of AEG-1 as a clinical biomarker of NSCLC. And we found that high expression of AEG-1 showed more robust correlation with high clinical stage, lymph node metastasis, distant metastasis, poorly histological differentiation and short overall survival (OS). However, some limitations and heterogeneity might be unavoidable in our meta-analysis. First, only published studies reported in English or Chinese were involved, which might result in missing some qualified papers according to language criteria. Second, since Sun et al. [14] did not apply the multivariate analysis directly, the relevant data required to be extracted from the Kaplan-Meier curve, which might lead to an inaccurate HR. Meanwhile, the different cut-off values for the AEG-1 SI in the studies and the small sample sizes of some studies were also factors to produce bias. Moreover, there were different quantitative methods, such as IHC and RT-PCR, which might generate inevitable clinical biases. Despite the limitations in this meta-analysis, our study still suggested that the AEG-1 level was significantly associated with tumor size, clinical stage and lymph node metastasis of NSCLC. Given the different AEG-1 positivity rates in NSCLC, it is necessary to further explore whether AEG-1 could be a predictive biomarker in patients with NSCLC. Generally, the clinical stage, status of tumor size, metastasis and overall survival could reflect the deterioration of a tumor [20, 21], so the high AEG-1 expression could be closely related to the deterioration of NSCLC.

Consistently with our results in the TCGA database and meta-analysis, the results from the tissue microarray demonstrated that AEG-1 was overexpressed in NSCLC compared with normal lung, and there was a significant correlation between AEG-1 expression and tumor size, clinical stage and lymph node metastasis, which are all in agreement with the previous findings of Ren et al. [16]. Furthermore, the high AUC of AEG-1 indicates that AEG-1 might be a significant molecule for diagnosing NSCLC. To further explore the biological role of AEG-1 expression *in vitro* and *in vivo*, the results clearly confirmed that AEG-1 was mostly overexpressed in the H460 cells. Next, shRNA was transfected to silence the expression of AEG-1, and the sh00 group showed the most effective suppression. We also performed direct experimental evidence by scratch assay, colony formation assay, Transwell migration and invasion assay and the CAM model to demonstrate that AEG-1 was a tumorigenic gene and that the high expression of AEG-1 promotes the proliferation, invasion and migration of the NSCLC cells.

To date, several studies have reported the effect and potential mechanism of AEG-1 on NSCLC. Zhu et al. [22] found that AEG-1 was an oncogene and highly expressed in NSCLC tissues, and AEG-1 could promote the invasiveness of NSCLC cells via up-regulating MMP7 levels as assessed by RT-qPCR, Western blot and ELISA. Their data also suggested that AEG-1 might function upstream of MMP7, and AEG-1 might increase MMP7 expression via MAPK-p42/p44 signaling pathway. Ke et al. [23] found that a high expression of AEG-1 was related to clinical staging, differentiation, lymph node metastasis and overall survival. Also, they applied MTT, TUNEL, flow cytometry assay and immunohistochemistry to verify that AEG-1 could play a pivotal role in the carcinogenesis of NSCLC and could suppress apoptosis via activating cell survival signaling, e.g., improving the level of anti-apoptotic protein Bcl-2 and the activation of PI3K/Akt pathway. However, in contrast to the aforementioned two studies, Yao et al. [15] found that a low AEG-1 expression was related to lymph node metastasis, TNM stage and overall survival by analyzing 40 NSCLC patients. And they found that increasing expression of AEG-1 could inhibit proliferation, tumour formation and metastasis of NSCLC cells via actin cytoskeletal remodeling. In comparison, we specifically designed this study using *in vitro* and *in vivo* experiments to further explore the effect of AEG-1 in NSCLC. Interestingly, we confirmed that AEG-1 was a tumorigenic gene and that a high expression of AEG-1 promotes the proliferation, invasion and migration of NSCLC cells. Based on the results of GO and KEGG analyses, we found that the most strongly enriched functional terms were protein processing, protein transport, protein export, and so on. Also, the AEG-1 co-expressed genes were significantly related to AMPK signaling pathway. As reported, Song et al. [24] found that AEG-1 can promote glycolysis and tumorigenesis in colorectal carcinoma cells via AMPK signaling. Also, several studies have revealed that AMPK signaling pathway played a significant role in energy metabolism and NSCLC [25–27]. In our current study, we hypothesized that AEG-1 could play an important role in NSCLC via AMPK signaling pathway, but the authentic underlying mechanism of AEG-1 in NSCLC still needs to be investigated with a further research.

## MATERIALS AND METHODS

### Study design on clinical samples

A tissue microarray was constructed in this study, which contained 339 NSCLC and 30 normal lung tissues. All of these lung cancer tissues were gathered from the First Affiliated Hospital of Guangxi Medical University, P. R. China (from January 2010 to February 2014). All of these cases were collected randomly from surgical resection without treatment. The study protocol was approved by the Ethical Committee of the First Affiliated Hospital of Guangxi Medical University, and the informed consent was provided from the clinicians and patients for the usage of the tissues for research. The mean age of these lung cancer and normal lung cases was 57.67 and 54.03 years, respectively. Among all of the NSCLC tissues, 127 cases were adenocarcinomas and 175 cases were squamous cell carcinomas. All of these tissues were evaluated and diagnosed for AEG-1 expression by two pathologists independently without knowing the patient information. Furthermore, the relationship between AEG-1 expression and the clinical diagnostic value was analyzed by an ROC curve [28]. The differential expression of AEG-1 in lung cancer and normal lung, in lung adenocarcinoma and squamous cell carcinoma was further explored.

### Evaluation of immunostaining

The expression of AEG-1 was detected by immunohistochemistry. The AEG-1 antibody was purchased from Cell Signaling Technology, Inc., China. The immunohistochemical staining reagents were supplied by Shanghai ChangDao Biotech Company, China. The immunohistochemistry procedure followed the manufacturer's protocol. Two independent pathologists scored the average percentage of positive cells as follows: 0 (0%); 1 (1–25%); 2 (26–50%); 3 (51–75%); and 4 (76–100%). The intensity of the staining was scored as follows: 0 (negative); 1 (weak); 2 (moderate) and 3 (strong). The final pathological scores were calculated by multiplying the percentage and the staining intensity score as previously described [29, 30]. The positive staining results were confirmed when the scores were over two.

### Additional analysis of AEG-1 in NSCLC from TCGA

TCGA (http://cancergenome.nih.gov/) is a collection of miRNA-seq, RNA-seq, SNP array, DNA methylation, exome sequencing and more [31]. TCGA can also be performed to analyze the complicated cancer genomics and clinical parameters [32, 33]. In this study, the original data of RNASeqV2 in lung adenocarcinoma and squamous cell carcinoma for AEG-1 were extracted from TCGA, and the expression levels of AEG-1 in each case was calculated according to the distribution of the exon reads. Then, the clinical diagnostic value of AEG-1 was analyzed by an ROC curve.

### AEG-1 and NSCLC: a meta-analysis

The data search was up to September 30, 2016. An extensive search of PubMed, Web of Science, Science Direct, Google Scholar, Ovid, LILACS, Wiley Online Library, EMBASE, Cochrane Central Register of Controlled Trials, Chong Qing VIP, Chinese CNKI, Wan Fang and China Biology Medicine disc was performed without language restrictions. The following keywords were searched: (metadherin OR MTDH OR astrocyte elevated gene-1 OR AEG1 OR AEG-1 OR LYRIC OR 3D3) AND (Cancer OR carcinoma OR adenocarcinoma OR tumour OR tumor OR malignanc* OR neoplas*) AND (Lung OR pulmonary OR respiratory OR respiration OR aspiration OR bronchi OR bronchioles OR alveoli OR pneumocytes OR “air way”). Reference lists from conference papers, clinical guidelines and relevant systematic reviews were also examined. Additionally, trails using either cell lines or animals, case reports, reviews and letters were excluded. In this study, only English and Chinese language publications were included. Two independent investigators (Yu Zhang and Xiao Wang) performed the literature retrieval and assessed the final included articles. The extracted data included the name of the first author, the year of the publication and the number of AEG-1 high expression and low expression in the age, gender, histological differentiation (poorly/well-moderately), clinical stage I/II and clinical stage III/IV, lymph node metastasis and non-metastasis, distant metastasis and non-metastasis groups.

### Cell culture and transfection with shRNA plasmid

The four human NSCLC cell lines (H460, H1299, A549, and PC9) were from the American Type Culture Collection (ATCC, Rockville, MD, USA). These four cell lines were cultured in Dulbecco's modified essential medium (DMEM) with 10% heat-inactivated fetal bovine serum (Invitrogen Corp, Grand Island, NY, USA). All of the NSCLC cells were maintained under a humidified 5% CO_2_ atmosphere at 37°C. Five shRNA plasmids were synthesized by GenePharma in Shanghai and were merged into one shRNA pool and the sequences of shRNA plasmids were shown in Table 8. Three groups were designed in this study as follows: the mock control, the negative shRNA control (NC) and the shRNA. The mock control groups were added with only transfection reagent. The negative control groups were treated with a negative shRNA (con77, GenePharma, Shanghai), and LipoFiter^TM^ (Hanbio, Shanghai) was used for the transfection on the manufacturer's instructions. In addition, after incubation for 24h, puromycin (2ug/ml) was added to select stable cell lines after transient transfection of shRNA plasmid. Then the transfection efficiency was evaluated under fluorescence microscope and RT-qPCR.

**Table 8:** The sequences of shRNA plasmids

shRNA	ID	Target Seq
sh97	AEG-1-RNAi (9997)	AAGTCAAATACCAAGCAAA
sh98	AEG-1-RNAi (9998)	ATGATGAATGGTCTGGGTT
sh99	AEG-1-RNAi (9999)	AACTACAACCGCATCATT
sh00	AEG-1-RNAi (10000)	CTTATTAATGGACAGCTTT
con77	Negative siRNA control	TTCTCCGAACGTGTCACG

### Cell block

In our study, we demonstrated the usefulness of NSCLC cytology with the cell block method based on the published studies [34, 35]. As reported, the cell block, combined with immunostaining, helps in the diagnosis and individualized treatment of cancer patients [36–38]. After centrifuging the NSCLC cell suspension for 3 min at 2,000×g, the supernatant was discarded. These preparations were then centrifuged for 5 min at 3,000×g. Afterwards, they were moved into an EP tube and were then centrifuged for 10 min at 3,500×g after adding ethanol. After that, the sediment was carefully placed in 10% formalin overnight at room temperature. The cell block was then taken out and was dehydrated with ethanol and embedded in paraffin. Sections of 3–5 μm in thickness were obtained for hematoxylin/eosin (HE) staining and immunohistochemistry.

### Western blot analysis

Western blot is widely used in biochemistry and molecular biology [39, 40]. In this study, Western blot was used in detecting the expression of AEG-1 protein in different cell lines and different groups. The NSCLC cells were washed with PBS in a 6-well-plate and were lysed in a buffer of 10% glycerol, 5% SDS, 5% 2-mercaptoethanol, 0.2% romophenol blue, 80 mM Tris-HCl (ph 6.8), 5 mM EDTA (ph 8) and 1 mM phenylmethylsulfonyl fluoride. Then, the lysates were centrifuged at 12,000×g for 10 min at 4°C and were boiled for 5 min. Hybond ECL nitrocellulose membranes (GE Healthcare Bio-sciences/Amersham, Diegem, Belgium) were applied to separate the proteins for 2 h at 100 mA. The immunoblot analyses were executed using the following antibodies: AEG-1 (Cell Signaling Technology) and GAPDH (Vazyme Biotech Co., Ltd). The image pro plus 6.0 was used to analyze the IOD value.

### RNA extraction and qRT-PCR

qRT–PCR was performed as reported [41]. Total RNA from the different cell groups were extracted using the Trizol reagent (Tiangen, Beijing) according to the kit instructions. The RNA extracted from each sample was used for complementary DNA synthesis through the SuperScript first strand cDNA synthesis kit (Vazyme Biotech Co., Ltd.) as stated by the manufacturer's protocol. In addition, qRT-PCRs were performed using a LightCycler 96 (Roche, Shanghai). The primer pairs applied for PCR were as follows: AEG-1, 5′-GAATCTCCGGAGCGAGGAAC-3′(forward); 5′-GGTGGCTGCTTTGCTGTTAC-3′ (reverse); GAPDH, 5′-CTGGCATTGGGTCTACTGCT-3′ (forward); 5′-GTCTACCCAATTGCCCCACT-3′ (reverse). The PCR cycle included 95°C for 10 minutes, 95°C for 15 seconds, and 95°C for 1 minute. The expression data were normalized to GAPDH to reduce the variability in the expression levels. In addition, standard curve method was used to draw standard curves to evaluate the stability and specificity of our primers and PCR system, then the quantification of gene expression was analyzed by Delta Delta Ct method [41, 42].

### Colony formation assay

To evaluate the proliferation of the 3 different cell groups (mock, NC, shRNA), a colony formation assay in soft agar was used as reported [43, 44]. The 6-well-plates were coated with 0.5% agar and RPMI-1640 plus 10% FBS at the bottom layer. Then, 500 cell/plate were seeded into the 6-well-plate. After a 7 day incubation at 37°C in 5% CO_2_, the colonies were fixed with 4% paraformaldehyde (PFA) and were stained with 0.005% crystal violet. The images were counted and visualized under a light microscope.

### Scratch assay

To further observe the effect of the AEG-1-shRNA on the migration of NSCLC, scratch assay was performed as reported [45, 46]. Exponentially growing cells (5×10^5^ cells/mL) were seeded into six-well-plate. The 3 different groups (mock, NC, shRNA) were incubated with under a humidified atmosphere of 5% CO_2_ at 37°C. When the cells spread to more than 80% of six-well-plate, 4 vertical lines were drawn with a sterile spear head (10μL) in each hole. Then the images were taken directly after scratching at 0 h, 12 h, 24 h and 48 h. The migration distance was quantized by Image-Pro Plus 6.0. The total distance was calculated by converting the pixels to millimeters.

### Transwell migration and invasion assay

To explore whether the expression of AEG-1 affected cell migration and invasion, Transwell migration and invasion assays were performed [40, 47]. For the migration assay, exponentially growing cells (5 × 10^5^ cells/mL) were seeded into the upper chamber (BD Bioscience) in culture medium with 1% FBS, whereas for the invasion assay, the upper chamber was pre-coated with Matrigel (BD Bioscience) prior to adding the cells (5 × 10^5^ cells/mL), and then they were all incubated for 24 h at 37°C. The lower chamber was added with 600 μL RPMI 1640 containing 10% FBS. Then, the cells on the upper surface of the filters would be scraped off with swabs. After fixed the membrane with 4% paraformaldehyde and stained with crystal violet, the cells were taken photos under microscope. At least 5 microscopic fields were counted for each transwell filter.

### CAM model of NSCLC

Fertilized chicken eggs were obtained from a local hatchery and were placed in a 10°C atmosphere before incubation at 37°C. After 8 days of incubation, NSCLC cells from the different groups were engrafted into the chick embryo chorioallantoic membrane (2.5 × 10^6^ cells per egg). Five chicken eggs were included in each group. Then, embryo viability and tumor growth were observed daily under the SZ61 Zoom Stereo Microscope (Olympus, Japan). Five days later, the tumor xenografts were removed from the CAMs, paraformaldehyde-fixed and paraffin-embedded [48]. The images of the tumor xenografts were obtained by an anatomy microscope. The size of the tumor xenografts was measured, and the tissue blocks were further observed by microscopic examination paraffin section. The size would be labeled 0 if the length of the tumor xenograft was less than 0.2 cm.

### The potential pathways associated with AEG-1

To further analyze the co-expressed genes and the underlying functions and pathways associated with AEG-1, an open-access resource, Multi Experiment Matrix (MEM, http://biit.cs.ut.ee/mem/index.cgi), was used to further explore the co-expressed genes of AEG-1 based on Affymetrix Gene Chip Human Genome U133 Plus 2.0 Array platform [49]. Then, bioinformatics analyses including GO, KEGG and network analysis, were performed to investigate the potential functions, pathways and networks of the co-expressed genes as described [50]. In this process, Database for Annotation, Visualization and Integrated Discovery (DAVID: available online: http://david.abcc.ncifcrf.gov/) was used to perform GO and KEGG analyses. And three independent categories would be derived from GO analysis: biological process (BP), cellular component (CC) and molecular function (MF). Besides, a functional network was constructed using Cytoscape (version 2.8, http://cytoscape.org). The final co-expressed genes were identified and compared using Venn diagrams (available online: http://bioinformatics.psb.ugent.be/webtools/Venn/).

### Statistical analysis

The AEG-1 expression between the two samples was evaluated by Student's t test and chi square tests. The comparison between groups was performed using one-way analysis of variance (ANOVA). For non-normally distributed variables, we performed Mann-Whitney *U* test and Kruskal–Wallis H test. The summary statistics were presented as mean ± standard deviation (Mean ± SD). The relationships between AEG-1 expression and the clinicopathological parameters were assessed by Spearman's rank correlation. The ROC curve was performed to detect the diagnostic value of AEG-1 expression in NSCLC tissue and cells. Mining for co-expression across hundreds of datasets was performed using novel rank aggregation and visualization methods. A *P* < 0.05 was regarded to be statistically significant (two sides) with SPSS 20.0. In addition, all the experiments were operated 6 repetitions.

In the meta-analysis, all of the statistical analysis were completed by STATA 12.0 (STATA Corp., College, Texas). Heterogeneity between the included studies was assessed by a χ^2^-based Q test and I^2^ tests, and I^2^ (%)>50% or *P* < 0.05 was regarded as significantly heterogeneous. Publication bias was evaluated by Begg's or Egger's test and P<0.05 indicated a statistical publication bias. The fixed effect model (Mantel-Haenszel) was applied to estimate the pooled odds ratios (ORs). In addition, to confirm whether the results were just due to a study with an extreme result or a large number, a sensitivity analysis was used to exclude one study at a time.

## CONCLUSIONS

Our findings confirm that overexpression of AEG-1 can promote the proliferation, invasion and migration of NSCLC and the high AEG-1 level can accelerate tumorigenesis and the deterioration of NSCLC. We also hypothesize that AEG-1 may play an important role in NSCLC via AMPK signaling pathway, but the authentic underlying mechanism of AEG-1 in NSCLC still needs to be investigated with a further research.
