# Correction: Clinical significance and effect of AEG-1 on the proliferation, invasion, and migration of NSCLC: a study based on immunohistochemistry, TCGA, bioinformatics, *in vitro* and *in vivo* verification

**DOI:** 10.18632/oncotarget.28212

**Published:** 2022-03-09

**Authors:** Yu Zhang, Zu-Yun Li, Xin-Xi Hou, Xiao Wang, Yi-Huan Luo, Yan-Ping Ying, Gang Chen

**Affiliations:** ^1^Department of Pathology, First Affiliated Hospital of Guangxi Medical University, Nanning, Guangxi Zhuang Autonomous Region 530021, China; ^2^Department of Orthopedics, China-Japan Union Hospital of Jilin University, Changchun 130033, China; ^3^Department of Nursing, The First Affiliated Hospital of Guangxi Medical University, Guangxi Zhuang Autonomous Region 530021, China; ^*^These authors contributed equally to this work

**This article has been corrected:** In Figure 1, the red boxes in panel “E” and “G” were not precisely matched to the enlarged areas of panel “F” and “H”. The areas have now been accurately redrawn in panel “E” and “G” to match with “F” and “H”. Two extra lines were also added to make the pictures easier to follow. Additionally, in Figure 11, the image of the NC group contains an accidental overlap of the image from the Mock group. The corrected Figures 1 and 11, produced from the original data, are shown below. The authors declare that these corrections do not change the results or conclusions of this paper.

Original article: Oncotarget. 2017; 8:16531–16552. 16531-16552. https://doi.org/10.18632/oncotarget.14972

**Figure 1:**
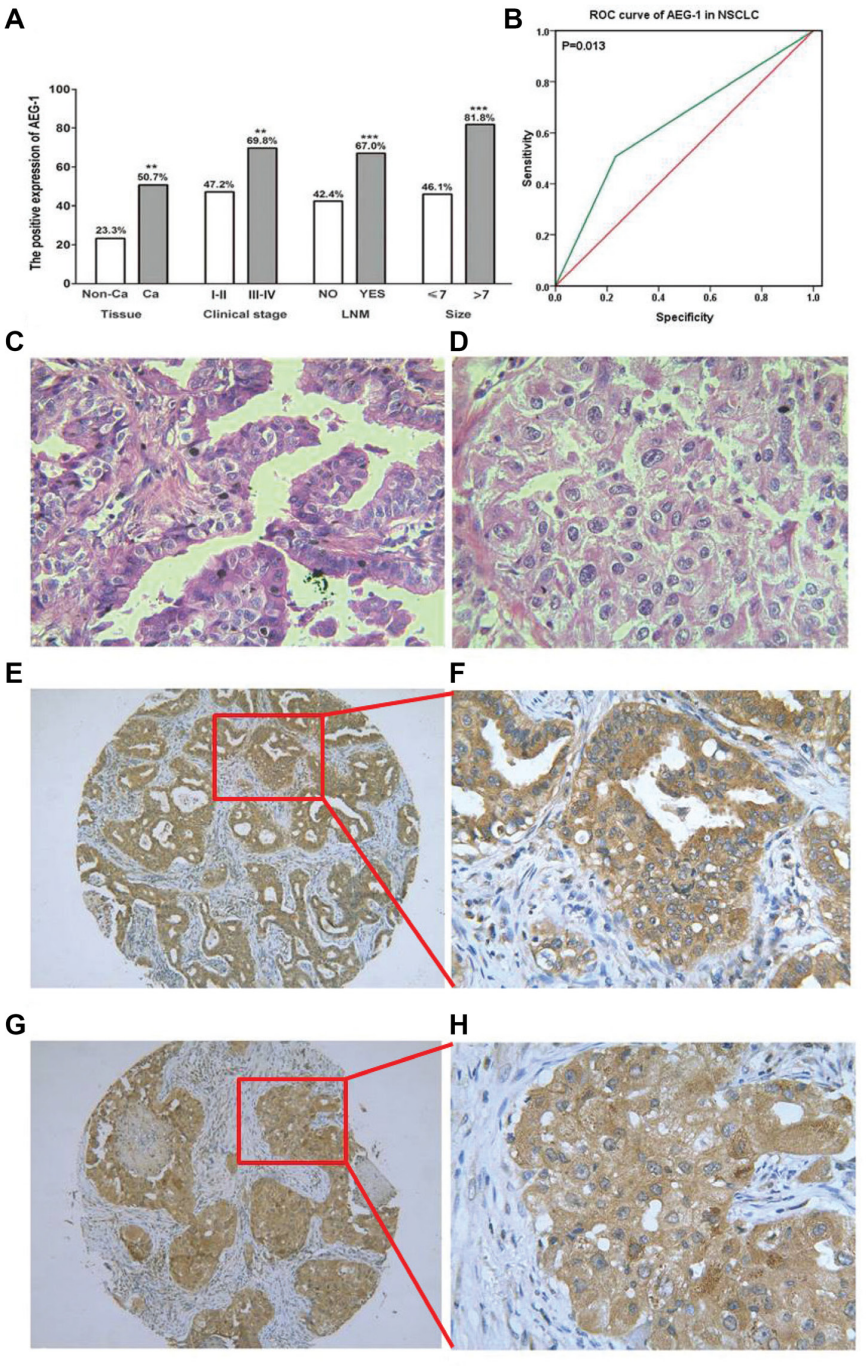
The relationship between AEG-1 expression and NSCLC. (**A**) The differential expression of AEG-1 between lung cancer and normal lung tissues, and the correlation between AEG-1 expression and clinical stage, LNM and tumor size (^**^ *P* < 0.01, ^***^ *P* < 0.001). Note: LNM: lymph node metastasis. (**B**) ROC curve analyses of AEG-1 for predicting the clinical diagnostic value in NSCLC. The area under curve (AUC) of AEG-1 was 0.637 (95% CI 0.540–0.734, *P* = 0.013), which indicates a potential diagnostic value of AEG-1 level in NSCLC. (**C**) Hematoxylin/eosin (HE) staining of lung adenocarcinoma tissues with AEG-1 expression (×400). (**D**) Hematoxylin/eosin (HE) staining of squamous cell carcinoma with AEG-1 expression (×400). (**E**) Immunohistochemical staining for AEG-1 in lung adenocarcinoma (×100). (**F**) Immunohistochemical staining for AEG-1 in lung adenocarcinoma (×400). (**G**) Immunohistochemical staining for AEG-1 in squamous cell carcinoma (×100). (**H**) Immunohistochemical staining for AEG-1 in squamous cell carcinoma (×400).

**Figure 11:**
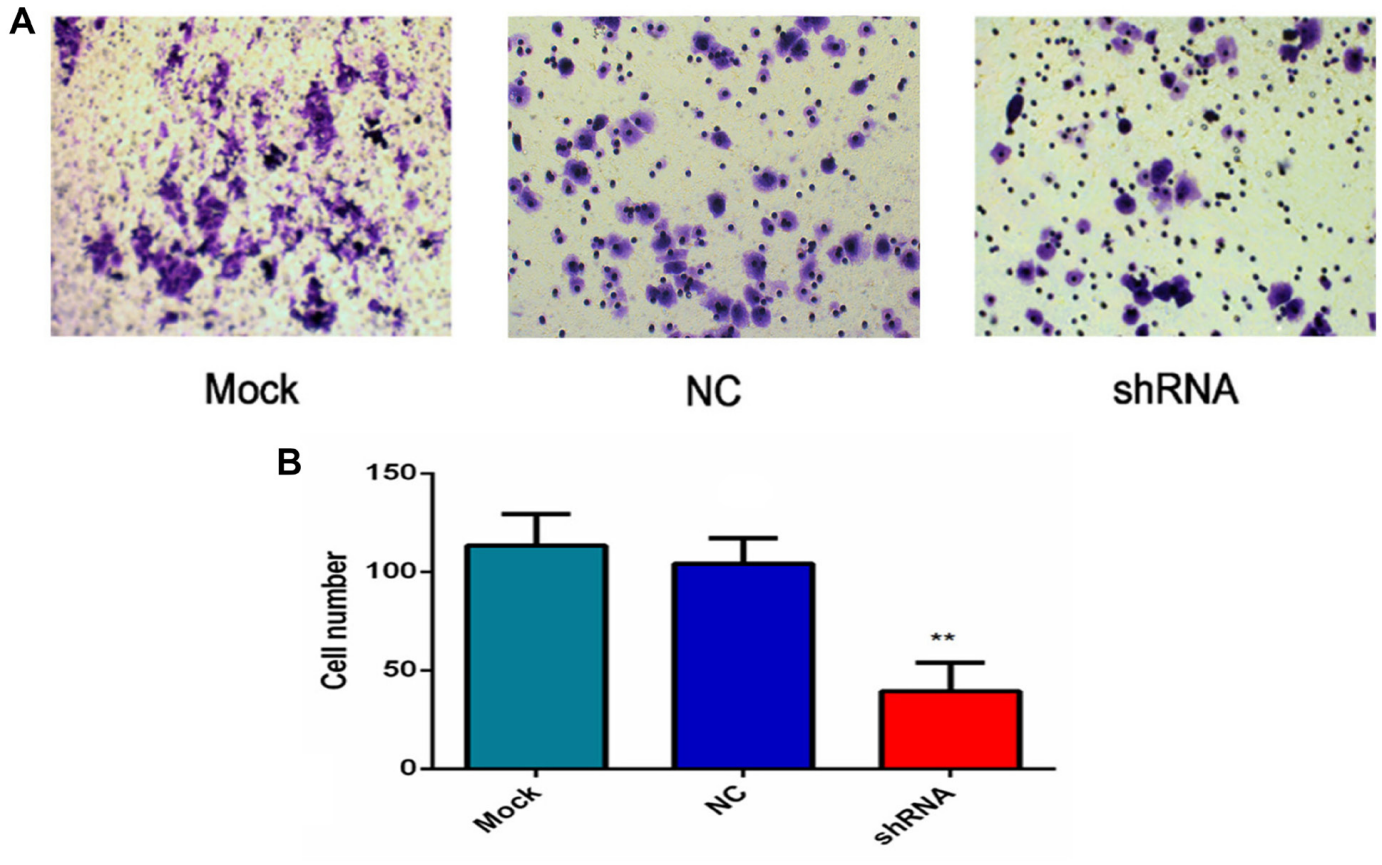
AEG-1-shRNA inhibited the invasion of the H460 cells. (**A**) Transwell invasion assay was performed to explore whether the expression of AEG-1 affected cell invasion, and the least number of the migratory cells was found in the shRNA group. (*P* < 0.01). (**B**) The number of invasive cells in the mock, NC and AEG-1-shRNA groups were counted. The graph represents the Mean ± SD. (^**^ *P* < 0.01).

